# The effect of umeclidinium added to inhaled corticosteroid/long-acting β_2_-agonist in patients with symptomatic COPD: a randomised, double-blind, parallel-group study

**DOI:** 10.1038/npjpcrm.2016.31

**Published:** 2016-06-23

**Authors:** Ana R Sousa, John H Riley, Alison Church, Chang-Qing Zhu, Yogesh S Punekar, William A Fahy

**Affiliations:** 1 GSK, Stockley Park, Uxbridge, Middlesex, UK; 2 GSK, Research Triangle Park, Durham, NC, USA; 3 GSK, Clinical Statistics (Respiratory), Stockley Park, Uxbridge, Middlesex, UK

## Abstract

Benefits of triple therapy with a long-acting muscarinic antagonist (LAMA), added to inhaled corticosteroid (ICS)/long-acting β_2_-agonist (LABA), have been demonstrated. Limited data assessing the efficacy of the LAMA umeclidinium (UMEC) added to ICS/LABA are available. The aim of this study is to evaluate the efficacy and safety of UMEC added to ICS/LABAs in patients with moderate-to-very-severe COPD. This is a multicentre, randomised, double-blind, parallel-group study. Patients were symptomatic (modified Medical Research Council Dyspnoea Scale score ⩾2), despite receiving ICS/LABA (fluticasone propionate/salmeterol (FP/SAL, branded) 500/50 mcg, budesonide/formoterol (BD/FOR, branded) 200/6 mcg or 400/12 mcg, or other ICS/LABAs) ⩾30 days before the run-in (7±2 days). Patients were randomised 1:1 to once-daily UMEC 62.5 mcg or placebo (PBO), added to twice-daily open-label ICS/LABA for 12 weeks. Primary end point was trough forced expiratory volume in 1 s (FEV_1_) at Day 85; secondary end point was weighted mean (WM) 0–6 h FEV_1_ at Day 84; other end points included COPD Assessment Test (CAT) score and Transition Dyspnoea Index (TDI) score. Adverse events (AEs) were investigated. In the UMEC+ICS/LABA and PBO+ICS/LABA groups, 119 and 117 patients were randomised, respectively. Patients received FP/SAL (40%), BD/FOR (43%) and other ICS/LABAs (17%). UMEC+ICS/LABA resulted in significant improvements in trough FEV_1_ (Day 85) and in WM 0–6 h FEV_1_ (Day 84) versus PBO+ICS/LABA (difference: 123 and 148 ml, respectively, both *P*<0.001). Change from baseline for UMEC+ICS/LABA versus PBO+ICS/LABA was significantly different for CAT score at Day 84 (−1.31, *P*<0.05), but not for TDI score (0.40, *P*=0.152). AE incidence was similar with UMEC+ICS/LABA (38%) and PBO+ICS/LABA (42%). UMEC+ICS/LABA improved lung function and CAT score in patients with symptomatic COPD versus PBO+ICS/LABA (ClinicalTrials.gov NCT02257372).

## Introduction

Chronic obstructive pulmonary disease (COPD) is characterised by persistent airflow limitation, which contributes significantly to morbidity and mortality, and it presents a significant economic burden worldwide.^1^ Recent estimates predict that COPD will be the third leading cause of death globally by 2030.^2^ Pharmacologic treatment of COPD is used to reduce symptoms, improve health status and exercise tolerance, and reduce the frequency and severity of exacerbations.^1^ Triple therapy (the combination of long-acting muscarinic antagonist (LAMA) with inhaled corticosteroid (ICS)/long-acting β_2_-agonist (LABA)) is recommended as a secondary treatment option in patients experiencing frequent COPD symptoms with a high risk of exacerbations.^1^ A recent retrospective study assessed trends in management and outcomes of COPD in general practice, including the use of triple therapy.^3^ The study showed that the use of triple therapy between 2004 and 2009 increased from 10 to 29% in moderate COPD, from 17 to 45% in severe COPD and from 25 to 59% in very severe COPD.^3^

Umeclidinium (UMEC) is a LAMA for which the 62.5 mcg (delivered dose, 55 mcg) dose has been approved as maintenance therapy for COPD in the US and the EU.^4,5^ Four Phase III, randomised studies have previously investigated the efficacy and safety of once-daily UMEC 62.5 mcg added to twice-daily fluticasone propionate/salmeterol (FP/SAL) 250/50 mcg or once-daily fluticasone furoate/vilanterol (FF/VI) 100/25 mcg.^6,7^ Statistically significant and clinically meaningful improvements in trough forced expiratory volume in 1 s (FEV_1_) were observed for UMEC 62.5 mcg added to FP/SAL 250/50 mcg or added to FF/VI 100/25 mcg compared with placebo (PBO) added to the respective ICS/LABA combinations (122−147 ml; *P*⩽0.001 for all). In all four studies, UMEC 62.5 mcg added to ICS/LABA was well-tolerated. *Post hoc* integrated analyses combining all four studies reported positive benefits on patient-reported outcomes (PROs) when using UMEC 62.5 mcg+ICS/LABA compared with PBO+ICS/LABA.^8,9^

FP/SAL and budesonide/formoterol (BD/FOR) are among the most widely used ICS/LABA combinations.^10^ Although the prospective data are available on the efficacy of UMEC added to FP/SAL 250/50 mcg^7^ and FF/VI,^6^ no studies have investigated the effect of UMEC added to other ICS/LABA combinations such as BD/FOR or the FP/SAL 500/50-mcg dose that is approved in the EU.^11^ As such, there is a need to determine the effect of UMEC added to other ICS/LABA combinations typically used to treat COPD, including FP/SAL 500/50 mcg and BD/FOR.

The purpose of the current add-on study was to evaluate the efficacy and safety of UMEC 62.5 mcg when added to frequently used ICS/LABA combinations at approved doses in patients with moderate-to-very-severe COPD who remained symptomatic despite receiving unselected ICS/LABA combinations before study entry. We believe that these results will complement and extend the pool of data already available for UMEC+ICS/LABA treatment, thus potentially increasing the generalisability of these data.

## Results

### Study population

**Figure 1:**
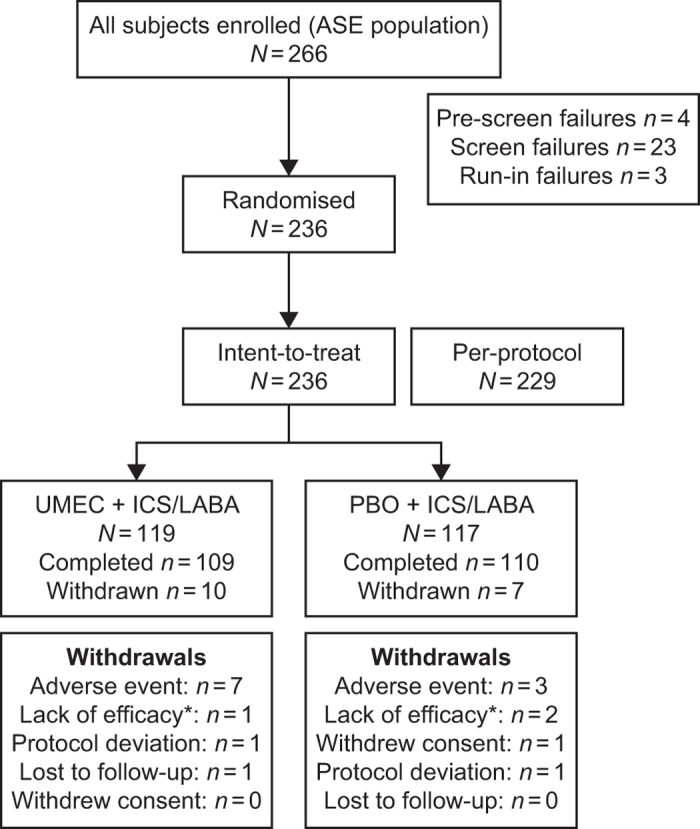
Patient disposition. *Lack of efficacy includes patients who withdrew because of COPD exacerbation; ASE, all subjects enrolled; COPD, chronic obstructive pulmonary disease; ICS, inhaled corticosteroid; LABA, long-acting β_2_-agonist; PBO, placebo; UMEC, umeclidinium.

A total of 266 patients were enrolled in the study. After screening, 236 patients were randomised and all were included in the intent-to-treat (ITT) population (Figure 1). A total of 229 patients in the ITT population were included in the per-protocol population. Overall, 219 (93%) patients in the ITT population completed the study (Figure 1).

In the UMEC+ICS/LABA and PBO+ICS/LABA groups, 119 and 117 patients were randomised, respectively, receiving FP/SAL 500/50 mcg (GSK, 40%), BD/FOR (AstraZeneca, 43%) and other ICS/LABA combinations including generics (17%) (Supplementary Table S1).

In terms of baseline characteristics, there was a smaller proportion of current smokers in the UMEC+ICS/LABA group than in the PBO+ICS/LABA group (49% versus 61%, respectively), but the number of smoking pack-years was similar in both treatment groups (Table 1). Similarly, there was a smaller proportion of high-risk (Global initiative for chronic Obstructive Lung Disease (GOLD) D) patients in the UMEC+ICS/LABA group than in the PBO+ICS/LABA group (Table 1). However, the proportion of patients with GOLD Stage II−IV COPD in each of the two treatment groups was similar (Table 1). The UMEC+ICS/LABA group experienced a smaller number of COPD exacerbations 12 months before screening than in the PBO+ICS/LABA group (28 and 43, respectively; Table 1).

### Outcomes

#### Primary end point

**Figure 2:**
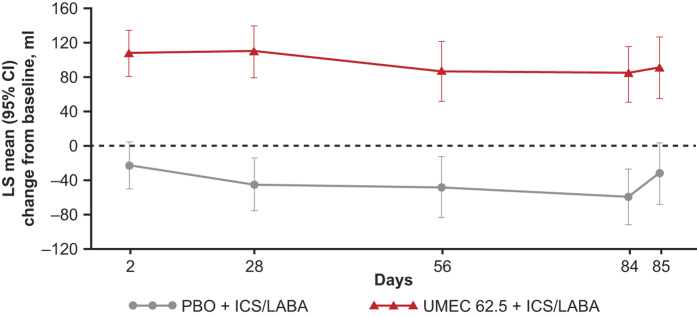
Least-squares mean (95% CI) change from baseline in trough FEV_1_ (ITT population). CI, confidence interval; FEV_1_, forced expiratory volume in 1 s; ICS, inhaled corticosteroid; ITT, intent-to-treat; LS, least squares; LABA, long-acting β_2_-agonist; PBO, placebo; UMEC, umeclidinium.

Compared with PBO+ICS/LABA, UMEC+ICS/LABA resulted in statistically significant and clinically meaningful improvements in change from baseline in trough FEV_1_ at Day 85 (123 ml, 95% confidence interval (CI): 71, 174; *P*<0.001; Table 2; Figure 2).

#### Secondary end points

UMEC+ICS/LABA resulted in statistically significant and clinically meaningful improvements in change from baseline in 0−6 h post-dose WM FEV_1_ at Day 84 compared with PBO+ICS/LABA (148 ml, 95% CI: 99, 197; *P*<0.001; Table 2).

#### Other end points

Statistically significant improvements in trough FEV_1_ were also observed at all other visits for UMEC+ICS/LABA compared with PBO+ICS/LABA (130 (95% CI: 91, 169)−154 ml (95% CI: 111, 198); *P*<0.001 for all; Figure 2).

Improvements for 0–6 h post-dose WM FEV_1_ similar to those observed at Day 84 were also observed at Days 1 and 28 for UMEC+ICS/LABA compared with PBO+ICS/LABA (123 ml, 95% CI: 89, 157; and 178 ml, 95% CI: 128, 228, respectively; *P*<0.001 for both days).

Forty-six percent of patients treated with UMEC+ICS/LABA had an increase in trough FEV_1_ of ⩾100 ml above baseline at Day 85 compared with 16% of patients treated with PBO+ICS/LABA (odds ratio (OR): 4.8, 95% CI: 2.6, 9.1; *P*<0.001; Table 2). The proportion of patients achieving an increase in FEV_1_ ⩾12% and ⩾200 ml in the first 6 h post dose on Day 1 was 56% and 24% in the groups UMEC+ICS/LABA and PBO+ICS/LABA, respectively (OR: 4.2, 95% CI: 2.4, 7.4;*P*<0.001; Table 2).

Statistically significant and clinically meaningful improvements in the change from baseline in FEV_1_ were observed at 15 min post dose on Day 84 for UMEC+ICS/LABA compared with PBO+ICS/LABA (127 ml, 95% CI: 75, 179; *P*<0.001). These improvements were maintained at 6 h post dose on Day 84 (156 ml, 95% CI: 107, 206; *P*<0.001; Supplementary Figure S1). Similar improvements were seen in the change from baseline in FEV_1_ with UMEC+ICS/LABA compared with PBO+ICS/LABA at 15 min post dose and 6 h post dose on Day 1 (66 ml; 95% CI: 40, 92; and 144 ml; 95% CI: 104, 185; respectively; *P*<0.001 for both), and at 15 min post dose and 6 h post dose on Day 28 (152 ml, 95% CI: 101, 203; and 180 ml, 95% CI: 123, 238; respectively; *P*<0.001 for both).

Statistically significantly greater change from baseline in peak FEV_1_ was demonstrated for UMEC+ICS/LABA compared with PBO+ICS/LABA at Day 84 (152 ml, 95% CI: 99, 205; *P*<0.001; Table 2). Similar improvements in peak FEV_1_ with UMEC+ICS/LABA compared with PBO+ICS/LABA were observed at Day 1 (125 ml, 95% CI: 89, 160; *P*<0.001) and Day 28 (180 ml, 95% CI: 128, 232; *P*<0.001).

Statistically significantly greater change from baseline in trough forced vital capacity (FVC) was demonstrated with UMEC+ICS/LABA compared with PBO+ICS/LABA at Day 85 (177 ml, 95% CI: 88, 267; *P*<0.001; Table 2). Similar improvements in trough FVC in change from baseline with UMEC+ICS/LABA compared with PBO+ICS/LABA were observed at Days 2, 28, 56 and 84 (165 (95% CI: 73, 257)−242 ml (95% CI: 165, 319); *P*<0.001 for all). Differences in serial FVC were statistically significantly greater for UMEC+ICS/LABA compared with PBO+ICS/LABA at all time points at Day 1 (113 (95% CI: 56, 170)−210 ml (95% CI: 136, 283); *P*<0.001 for all), Day 28 (196 (95% CI: 105, 287)−263 ml (95% CI: 166, 360); *P*<0.001 for all) and Day 84 (187 (95% CI: 87, 288)−234 ml (95% CI: 138, 329); *P*<0.001 for all).

**Figure 3:**
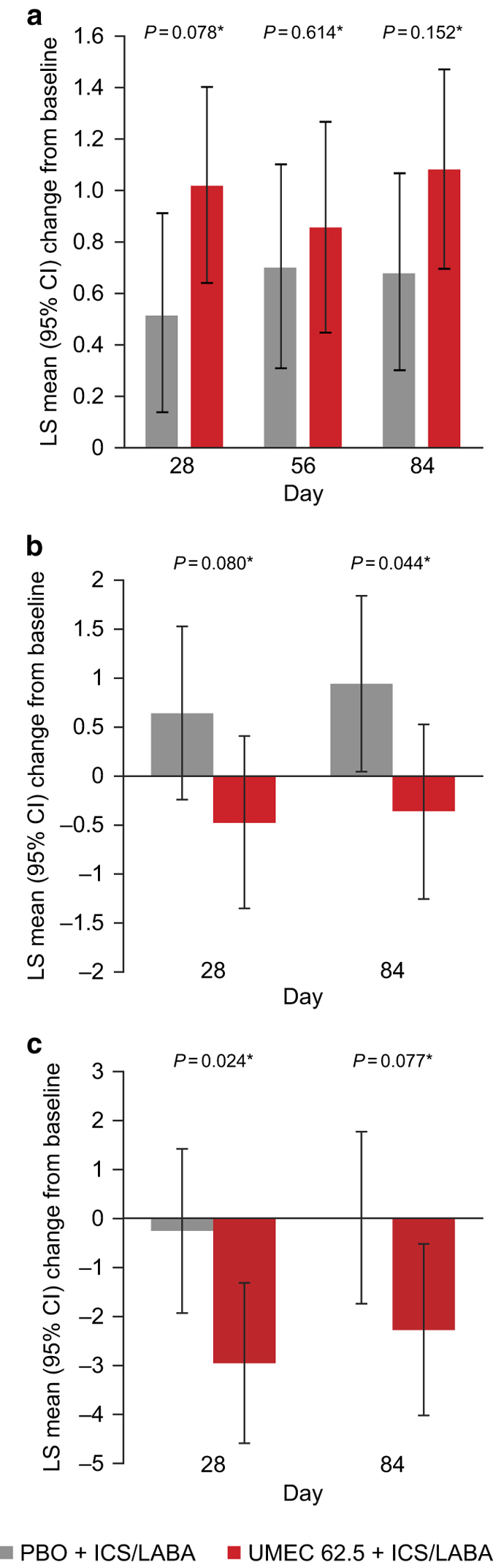
Least-squares mean (95% CI) change from baseline in (**a**) TDI score, (**b**) CAT score and (**c**) SGRQ score (ITT population). *P-values for treatment differences; CAT, COPD Assessment Test; CI, confidence interval; ICS, inhaled corticosteroid; ITT, intent-to-treat; LS, least squares; LABA, long-acting β_2_-agonist; PBO, placebo; SGRQ, St George’s Respiratory Questionnaire; TDI, Transition Dyspnoea Index; UMEC, umeclidinium.

For rescue medication use, UMEC+ICS/LABA resulted in a statistically significant reduction in change from baseline in mean puffs per day over weeks 1–12 versus PBO+ICS/LABA (−0.38, 95% CI: −0.67, −0.10; *P*<0.05; Table 2). However, no statistically significant difference was observed for percentage of rescue-free days for UMEC+ICS/LABA versus PBO+ICS/LABA (GSK data on file). Transition Dyspnoea Index (TDI) score at Day 84 was not significantly different for UMEC+ICS/LABA versus PBO+ICS/LABA (0.40, 95% CI: −0.15, 0.95; *P*=0.152; Table 2; Figure 3a). However, the mean TDI for the UMEC+ICS/LABA group exceeded the 1.0-unit threshold (1.07, s.e.: 0.197), which is considered the minimal clinically important difference for this end point,^12^ whereas the mean TDI in the PBO+ICS/LABA group (0.67, s.e.: 0.195) did not.

#### Health-related quality of life

Statistically significant improvements from baseline in COPD Assessment Test (CAT) score (where a negative number denotes an improvement) were observed at Day 84 for UMEC+ICS/LABA versus PBO+ICS/LABA (−1.31, 95% CI: −2.59, −0.04; *P*<0.05; Table 2; Figure 3b). UMEC+ICS/LABA resulted in statistically significantly greater odds of being a CAT responder at Day 84 (OR: 2.71, 95% CI: 1.52, 4.85; *P*<0.001; Table 2) compared with PBO+ICS/LABA. Change from baseline in SGRQ total score at Day 84 was not statistically significantly different for UMEC+ICS/LABA versus PBO+ICS/LABA (−2.26, 95% CI: −4.77, 0.25; *P*=0.077; Table 2; Figure 3c). UMEC+ICS/LABA did not result in a statistically significantly greater odds of being an SGRQ responder (OR: 0.96, 95% CI: 0.56, 1.67; *P*=0.892; Table 2).

#### Safety

The incidence of adverse events (AEs) was similar between the UMEC+ICS/LABA (38%) and PBO+ICS/LABA (42%) treatment groups (Table 3). The most common AEs were nasopharyngitis, 13 and 15%, and headache, 3 and 7%, in the UMEC+ICS/LABA and PBO+ICS/LABA groups, respectively. Pneumonia was reported for 3 (3%) patients and 2 (2%) patients in the UMEC+ICS/LABA and PBO+ICS/LABA groups, respectively. Tachycardia was reported for a total of 2 (2%) patients in the PBO+ICS/LABA treatment group and none in the UMEC+ICS/LABA group. No other individual AE of special interest was reported in >1 (<1%) patient in each treatment group.

The number of on-treatment non-fatal serious AEs was similar between the UMEC+ICS/LABA and PBO+ICS/LABA treatment groups (6 (5%) and 4 (3%), respectively; Table 3). The number of patients who experienced COPD exacerbations was balanced between the UMEC+ICS/LABA and PBO+ICS/LABA groups (17 (14%) and 16 (14%), respectively; Table 3). There was one (<1%) fatality in the PBO+ICS/LABA group; this was not drug-related. No fatalities were reported in the UMEC+ICS/LABA group.

#### *Post hoc* analysis of trough FEV_1_

In the *post hoc* subgroup analyses of trough FEV_1_ by ICS/LABA subgroup, UMEC+FP/SAL 500/50 mcg resulted in a statistically significant improvement in change from baseline in trough FEV_1_ at Day 85 versus PBO+FP/SAL 500/50 mcg (156 ml, 95% CI: 77, 235; *P*<0.001; *n*=42 in each treatment group). UMEC+BD/FOR also resulted in statistically significant improvement in change from baseline in trough FEV_1_ at Day 85 versus PBO+BD/FOR (130 ml, 95% CI: 55, 204; *P*<0.001; *n*=49 in each treatment group). No statistically significant difference was observed for change from baseline in trough FEV_1_ at Day 85 with UMEC added to other ICS/LABA combinations (50 ml, 95% CI: −106, 207; *P*=0.519; *n*=18 and *n*=19 in the UMEC+ICS/LABA and PBO+ICS/LABA groups, respectively).

## Discussion

### Main findings

As limited prospective data are available on the efficacy of UMEC added to ICS/LABA combinations, this study aimed to build on and expand the available data set on open-label triple therapy. This study demonstrated statistically significant and clinically meaningful improvements in trough FEV_1_ at Day 85 with UMEC+ICS/LABA compared with PBO+ICS/LABA in patients with moderate-to-very-severe COPD who remained symptomatic on a range of different ICS/LABAs. These ICS/LABA combinations included FP/SAL 500/50 mcg (GSK) and BD/FOR (AstraZeneca), as they are among the most widely used ICS/LABAs. Other ICS/LABA combinations, including generics, were also included. Significant improvements in 0–6 h post-dose WM FEV_1_ after 12 weeks of treatment were also observed.

All treatments in this study were well-tolerated, and the incidence of AEs was similar between the UMEC+ICS/LABA (38%) and PBO+ICS/LABA (42%) treatment groups. No additional safety concerns were identified with the addition of UMEC to ICS/LABA.

In a *post hoc* subgroup analysis of the primary end point of trough FEV_1_ at Day 85 by ICS/LABA type, statistically significant improvements in FEV_1_ were observed in the FP/SAL 500/50 mcg and BD/FOR subgroups but not in the smaller subgroup including other ICS/LABAs (including generics). The improvements observed in trough FEV_1_ with UMEC+FP/SAL 500/50 mcg and UMEC+BD/FOR were similar and were also consistent with those seen in previous studies of UMEC added to FP/SAL 250/50 mcg twice daily^7^ and UMEC added to FF/VI 100/25 mcg once daily.^6^ These findings suggest that the choice of background ICS/LABA therapy is unlikely to have any impact on the add-on efficacy of UMEC 62.5 mcg when used as triple therapy in patients who remain symptomatic on ICS/LABA therapy. The lack of a significant difference observed between UMEC+ ICS/LABA and the PBO+ICS/LABA for the other ICS/LABA subgroup may have been related to the small number of patients who received other ICS/LABA combinations in this study.

### Interpretation of findings in relation to previously published work

This study was specifically designed to examine the effect of the addition of UMEC to ICS/LABA on lung function. In addition, the study assessed the treatment difference in subjective PROs including TDI focal score and CAT score for the first time. However, the study was not formally powered to detect treatment differences in PRO measures. Despite the potential underpowering of this study to assess PROs, a statistically significant improvement was observed in CAT score after 12 weeks, with 20% more patients experiencing a clinically relevant improvement (⩾2-unit change^13^) in CAT score. The mean improvement in the TDI focal score showed a clinically relevant improvement in breathlessness (>1-unit change^12^) from baseline in the UMEC+ICS/LABA group but not in the PBO+ICS/LABA group. However, a statistically significant treatment difference for TDI score was not observed. The mean improvement in the total SGRQ score with UMEC 62.5 mcg triple therapy compared with ICS/LABA alone was statistically significant at Week 4, but not at Week 12; however, the magnitude of the treatment effect was similar at both visits and was in line with the other larger UMEC+ICS/LABA studies previously reported.^6^ As seen in other studies of UMEC+ICS/LABA, statistically significant reductions in the number of puffs per day of rescue medication use, an indirect measure of daily symptoms, were seen with UMEC+ICS/LABA versus PBO+ICS/LABA.^6–8^ UMEC monotherapy has been shown to provide meaningful improvements in PROs such as SGRQ.^14^ The improvements observed in this study were not consistent across PRO measures and did not reach reported minimal clinically important differences, possibly because all baseline PROs were measured while patients were receiving ICS/LABA.

### Strengths and limitations of this study

Strengths of the current study include the range of ICS/LABAs that patients were initially receiving, which has not been included in previous UMEC add-on studies.^4,6^ However, there are also limitations of this study that should be considered. The study was not powered to detect differences in end point results between treatment groups in the different ICS/LABA subgroups. Although the *post hoc* sensitivity analysis reached statistical significance for differences in trough FEV_1_ in UMEC+ICS/LABA versus PBO+ICS/LABA in two of the ICS/LABA types (FP/SAL 500/50 mcg and BD/FOR), which were relatively small samples, the number of patients in the other ICS/LABA subgroup was inconclusive because of very small sample sizes.

Finally, this study only assessed COPD exacerbations as a safety outcome over the relatively short 3-month follow-up period. The patients in this study may not have been at risk of exacerbation during the trial, as they did not require a history of exacerbation to enter the trial. Exacerbations were reported in a similar number of patients on both regimens as safety outcomes in the current analysis. A duration longer than 12 weeks, in a much larger population, would be recommended to assess the effect of UMEC added to ICS/LABA on COPD exacerbations as an efficacy end point. However, a large pooled integrated analysis of four previous UMEC 62.5 mcg+ICS/LABA 12-week trials, performed in patients with and without an exacerbation history, has demonstrated a benefit of UMEC triple therapy versus ICS/LABA alone in reducing the risk of a first moderate/severe COPD exacerbation.^8,9^

### Implications for future research policy and practice

Several placebo-controlled randomised studies have addressed the efficacy of UMEC in improving lung function and symptoms when given as a monotherapy^14,15^ or add-on therapy to ICS/LABA.^7,8^ However, future studies comparing UMEC with other LAMAs as monotherapy or in addition to ICS/LABA are warranted.

As the current study was not powered to detect differences in PRO measurements, further studies are needed to investigate the effect of UMEC on these measures.

Long-term exacerbation studies are also needed to document the benefits of UMEC-containing triple therapy compared with ICS/LABA therapy alone. The InforMing the PAthway of COPD Treatment (IMPACT; NCT02164513) exacerbation study, which is currently ongoing to evaluate the benefit of UMEC triple therapy versus both UMEC/VI or FF/VI, may answer these questions in a higher exacerbation risk COPD population.

### Conclusions

In summary, UMEC added to an existing choice of ICS/LABA improved lung function, rescue medication use and CAT score, one measure of HRQoL, when compared with PBO in symptomatic patients with COPD. No additional safety concerns were identified with UMEC in this add-on setting.

## Materials and methods

### Study design

This was a 12-week, multicentre, randomised, double-blind, parallel-group study conducted between September 2014 and March 2015 in the Czech Republic, Germany, Greece and the Netherlands (GSK study number: 201314; ClinicalTrials.gov registration number: NCT02257372).

The study protocol and informed consent were reviewed and approved by a national, regional or investigational centre ethics committee or institutional review board, in accordance with Good Clinical Practice (GCP). This study was conducted in accordance with GCP and the ethical principles outlined in the Declaration of Helsinki.

### Patients

Patients eligible for inclusion were ⩾40 years of age with an established clinical history of COPD, current or former smokers with ⩾10 pack-years smoking history, had a pre- and post-albuterol/salbutamol FEV_1_/FVC ratio of <0.7 and a pre- and post-albuterol/salbutamol FEV_1_ of ⩽70% of predicted normal values. Patients also had a dyspnoea score of ⩾2 on the modified Medical Research Council Dyspnoea Scale^16^ at Visit 1 and remained symptomatic after receiving one of the ICS/LABA combinations approved for COPD ⩾30 days before screening.

Exclusion criteria were a current diagnosis of asthma, hospitalisation for COPD or pneumonia within 12 weeks before Visit 1, lung volume reduction surgery within the 12 months before Visit 1, lower-respiratory-tract infection requiring antibiotic use within 6 weeks of Visit 1, use of long-term oxygen therapy (prescribed for >12 h per day) and participation in the acute phase of a pulmonary rehabilitation programme within 4 weeks before Visit 1. Additional exclusion criteria were evidence of concurrent respiratory disease or other clinically significant medical condition. This included an abnormal and clinically significant electrocardiogram finding at Visit 1 determined by atrial fibrillation with a rapid ventricular rate (>120 b.p.m.), ventricular tachycardia or second-degree heart block Mobitz type II or third-degree heart block (unless a pacemaker or defibrillator had been inserted). The use of prohibited medications (Supplementary Table S2) within the specified time periods also excluded patients from the study.

### Study treatments

Patients must have been receiving ICS/LABA at doses and frequencies approved for COPD ⩾30 days before the run-in period of 7±2 days. The ICS/LABA combinations received in this study were as follows: FP/SAL 500/50 mcg twice daily, BD/FOR 200/6 mcg twice daily or 400/12 mcg twice daily, or any other ICS/LABA combinations including generics listed in Supplementary Table S1. It should be noted that patients receiving FP/SAL 250/50 mcg or FF/VI 100/25 mcg were not included in the current study, as previous studies have investigated these. After the run-in period, patients were randomised 1:1 to UMEC 62.5 mcg (delivering 55 mcg) once daily or PBO once daily, added to their open-label ICS/LABA twice daily for 12 weeks. UMEC and PBO were double-blinded and administered via the ELLIPTA inhaler (Duhram, NC, USA).

Randomisation was stratified by the type of open-label ICS/LABA administered (FP/SAL, BD/FOR or other ICS/LABA combinations including generics). Randomisation codes were generated by GSK using a validated computerised system (RandAll NG). Patients were randomised using an Interactive Voice Response System.

### Outcomes

#### Primary end point

The primary efficacy end point was trough FEV_1_ on Day 85 (defined as the mean of the FEV_1_ values obtained 23 and 24 h after dosing on Day 84).

#### Secondary end points

The secondary end point was WM 0–6 h FEV_1_ at Day 84.

#### Other end points

Other efficacy end points included trough FEV_1_ and WM FEV_1_ over 0–6 h post dose at other time points, proportion of patients achieving an increase of ⩾100 ml above baseline in trough FEV_1_, proportion of patients achieving an increase in FEV_1_ of ⩾12% and ⩾200 ml above baseline at any time during 0–6 h post dose on Day 1, serial FEV_1_ over 0–6 h (at each time point); peak FEV_1,_ and trough and serial FVC. Rescue albuterol/salbutamol use (mean puffs per day and percentage of rescue-free days) and TDI focal score at Day 84 were also measured.

#### HRQoL

Health-related quality of life (HRQoL) end points included CAT score,^17,18^ proportion of CAT responders (defined as a reduction from baseline of ⩾2 units in CAT score), St George’s Respiratory Questionnaire for COPD patients (SGRQ-C) score and the proportion of SGRQ responders (defined by a reduction from baseline of ⩾4 units in SGRQ score).^19^ SGRQ scores were calculated from the SGRQ-C scores using standardised adjustment.

#### Safety

Safety assessments included monitoring of AEs, clinical laboratory tests, vital signs, physical examinations and COPD exacerbations. A COPD exacerbation was defined as an acute worsening of symptoms of COPD requiring the use of any treatment beyond study medication or rescue albuterol/salbutamol.

#### *Post hoc* analysis

A *post hoc* sensitivity analysis of trough FEV_1_ at Day 85 was conducted by the ICS/LABA subgroup. Three subgroups were defined: FP/SAL 500/50 mcg, BD/FOR and other ICS/LABA combinations including generics.

### Statistical analysis

Sample size calculations were based on the primary end point of trough FEV_1_ at Day 85. They assumed 90% power and a 2-sided 5% significance level with an estimate of residual standard deviation for trough FEV_1_ of 220 ml and a treatment difference of 100 ml. Under these assumptions, and to account for an estimated withdrawal rate of 10%, 115 evaluable patients on each treatment (230 in total) were required for 90% power to detect a 100 -ml difference between treatments in trough FEV_1_.

Three patient populations were identified: all patients enrolled population (all patients for whom a record existed on the study database), ITT population (all patients who were randomised to treatment and received at least one dose of randomised study medication) and per-protocol population (all patients in the ITT population who did not have a full protocol deviation considered to impact efficacy).

The UMEC+ICS/LABA versus PBO+ICS/LABA treatment comparison was performed on the primary end point. If statistical significance was demonstrated at the 5% level for the primary end point, then inferences on the secondary and other end points were made for the ITT population.

Primary end point trough FEV_1_ at Day 85 was analysed for the ITT population using a mixed model repeated measures analysis, including trough FEV_1_ recorded at each of Days 2, 28, 56, 84 and 85. The model included covariates of baseline FEV_1_, type of ICS/LABA (FP/SAL, BD/FOR or other), smoking status, day, treatment and day-by-baseline interaction, where day is nominal. A day-by-treatment interaction term was also included to allow treatment effects to be estimated at each visit separately.

The *post hoc* analyses of trough FEV_1_ at Day 85 by ICS/LABA subgroup were performed using a mixed model repeated measures analysis with covariates of treatment, baseline FEV_1_, smoking status, day, day-by-baseline and day-by-treatment interactions.

## Figures and Tables

**Table 1:** Patient baseline characteristics and demographics (ITT population)

	*UMEC+ICS/LABA (*N*=119)*	*PBO+ICS/LABA (*N*=117)*
Age, years	65.2 (7.5)	63.1 (7.9)
Male, *n* (%)	83 (70)	75 (64)
Current smoker at screening, *n* (%)	58 (49)	71 (61)
Smoking pack-years	45.6 (25.8)	44.0 (19.8)
Baseline FEV_1_ (l)	1.332 (0.486)	1.368 (0.500)
% predicted FEV_1_	47.6 (12.0)	47.8 (11.6)
Post-salbutamol FEV_1_/FVC	47.3 (10.5)	45.9 (10.1)
% reversibility to salbutamol^a^	8.6 (9.9)	10.1 (10.9)
Reversibility to salbutamol (ml)	100.4 (117.6)	124.1 (138.9)
		
*GOLD Stage using percent predicted FEV*_*1*_		
GOLD Stage II, *n* (%)	57 (48)	52 (44)
GOLD Stage III, *n* (%)	51 (43)	57 (49)
GOLD Stage IV, *n* (%)	11 (9)	8 (7)
		
*GOLD Category using the mMRC dyspnoea scale*		
GOLD Category B, *n* (%)	55 (46)	45 (38)
GOLD Category D, *n* (%)	64 (54)	72 (62)
		
Baseline salbutamol use (puffs per day)	1.6 (2.3)	1.7 (2.2)
Baseline CAT score	16.0 (6.9)	16.9 (6.3)
Baseline SGRQ total score	42.8 (15.3)	44.0 (14.9)
BDI focal score	6.3 (1.7)	6.2 (1.7)
Cardiovascular risk factors (any condition), *n* (%)	80 (67%)	74 (63%)
		
*Patients with COPD exacerbations in the 12 months before screening, n (%)*		
Managed without oral/systemic corticosteroids and/or antibiotics (without hospitalisation)	9 (8)	7 (6)
Requiring oral/systemic corticosteroids and/or antibiotics	19 (16)	31 (26)
Requiring hospitalisation	0 (0)	5 (4)

Values are reported as mean (s.d.), unless otherwise stated.

Abbreviations: BDI, baseline dyspnoea score; CAT, COPD assessment test; COPD, chronic obstructive pulmonary disease; FEV_1_, forced expiratory volume in 1 s; FVC, forced vital capacity; ICS, inhaled corticosteroid; LABA, long-acting β_2_-agonist; mMRC, modified medical research council; PBO, placebo; SGRQ total score, calculated based on St George’s Respiratory Questionnaire for COPD patients; TDI, Transition Dyspnoea Index; UMEC, umeclidinium.

a One patient in the UMEC 62.5+ICS/LABA treatment group did not have % reversibility to salbutamol recorded.

**Table 2:** Lung function end points (ITT population)

	*UMEC+ICS/LABA (*N*=119)*	*PBO+ICS/LABA (*N*=117)*	*Treatment Diff. versus PBO (95% CI)*	P*-value*
Trough FEV_1_ at Day 85	*n*=109	*n*=110		
LS mean change from baseline, ml (s.e.)	90 (18.3)	−33 (18.4)	123 (71, 174)	*P*<0.001
0–6 h weighted mean FEV_1_ at Day 84	*n*=107	*n*=110		
LS mean change from baseline, ml (s.e.)	184 (17.6)	35 (17.5)	148 (99, 197)	*P*<0.001
Proportion of patients with trough FEV_1_ ⩾100 ml above baseline at Day 85, *n* (%)	*n*=119 55 (46)	*n*=117 19 (16)	4.8 (2.6, 9.1)^a^	*P*<0.001
Proportion of patients with FEV_1_ increase ⩾12% and ⩾200 ml above baseline at Day 1, *n* (%)	*n*=119 67 (56)	*n*=117 28 (24)	4.2 (2.4, 7.4)^a^	*P*<0.001
Peak FEV_1_ at Day 84	*n*=110	*n*=110		*P*<0.001
LS mean change from baseline, ml (s.e.)	262 (18.8)	110 (18.9)	152 (99, 205)	
Trough FVC at Day 85	*n*=109	*n*=110		*P*<0.001
LS mean change from baseline, ml (s.e.)	99 (31.9)	−79 (32)	177 (88, 267)	
Rescue use (mean puffs per day)^b^	*n*=119	*n*=116		
LS mean change from baseline (s.e.)	−0.53 (0.11)	−0.15 (0.11)	−0.38 (−0.67, −0.10)	*P*<0.05
TDI score at Day 84	*n*=105	*n*=109		
LS mean (s.e.)	1.07 (0.20)	0.67 (0.20)	0.40 (−0.15, 0.95)	*P*=0.152
CAT score at Day 84	*n*=110	*n*=110		
LS mean change from baseline (s.e.)	−0.37 (0.46)	0.94 (0.46)	−1.31 (−2.59, −0.04)	*P*<0.05
CAT responders^c^ at Day 84	*n*=114	*n*=112		
Responder, *n* (%)	54 (47)	30 (27)	2.71 (1.52, 4.85)^a^	*P*<0.001
SGRQ score at Day 84	*n*=109	*n*=106		
LS mean change from baseline (s.e.)	−2.26 (0.89)	−0.00 (0.91)	−2.26 (−4.77, 0.25)	*P*=0.077
SGRQ responders^d^ at Day 84	*n*=119	*n*=114		
Responder, *n* (%)	42 (35)	43 (38)	0.96 (0.56, 1.67)^a^	*P*=0.892

Abbreviations: CAT, COPD assessment test; CI, confidence interval; COPD, chronic obstructive pulmonary disease; FEV_1_, forced expiratory volume in 1 s; FVC, forced vital capacity; ICS, inhaled corticosteroid; ITT, intent-to-treat; LABA, long-acting β_2_-agonist; LS, least squares; PBO, placebo; SGRQ total score, calculated based on St George’s Respiratory Questionnaire for COPD patients; TDI, Transition Dyspnoea Index; UMEC, umeclidinium.

a Odds ratio (95% CI).

b Use over 1–12 weeks.

c Response was defined as an improvement in CAT score of ⩾2.

d Response was defined as a reduction from baseline of 4 units in SGRQ score; improvements in CAT scores are shown by negative changes.

**Table 3:** Summary of on-treatment AEs

	*UMEC+ICS/LABA (*N*=119)*	*PBO+ICS/LABA (*N*=117)*
Any on-treatment AE, *n* (%)	45 (38)	49 (42)
		
*Most common on-treatment AEs reported by ⩾3% of patients in any treatment group by study, n (%)*		
Nasopharyngitis	16 (13)	17 (15)
Headache	4 (3)	8 (7)
Cough	3 (3)	5 (4)
Back pain	2 (2)	5 (4)
Pneumonia	3 (3)	2 (2)
Chest Pain	0	3 (3)
Diarrhoea	3 (3)	0
Oropharyngeal Pain	0	3 (3)
Upper Respiratory Tract Infection	3 (3)	0
		
Any on-treatment non-fatal drug-related SAEs, *n* (%)	0	0
Any on-treatment fatal drug-related SAEs, *n* (%)	0	0
Any on-treatment non-fatal SAEs, *n* (%)	6 (5)	4 (3)
Any on-treatment fatal SAEs, *n* (%)	0	1 (<1)^a^
Any on-treatment AEs leading to permanent discontinuation of medication/withdrawal, *n* (%)	7 (6)	3 (3)
Number of patients with a COPD exacerbation, n (%)	17 (14)	16 (14)

Abbreviations: AE, adverse event; COPD, chronic obstructive pulmonary disease; ICS, inhaled corticosteroid; LABA, long-acting β_2_-agonist; PBO, placebo; SAE, serious adverse event; UMEC, umeclidinium.

a There was one death in the group on Day 23; this was not drug-related (road traffic accident).
