# Supplementary figures and images for: The effect of umeclidinium added to inhaled corticosteroid/long-acting β2-agonist in patients with symptomatic COPD: a randomised, double-blind, parallel-group study
Source: NPJ Prim Care Respir Med. 2016 Jun 23;26:16031–. doi: 10.1038/npjpcrm.2016.31 (PMC4918053; doi:10.1038/npjpcrm.2016.31)

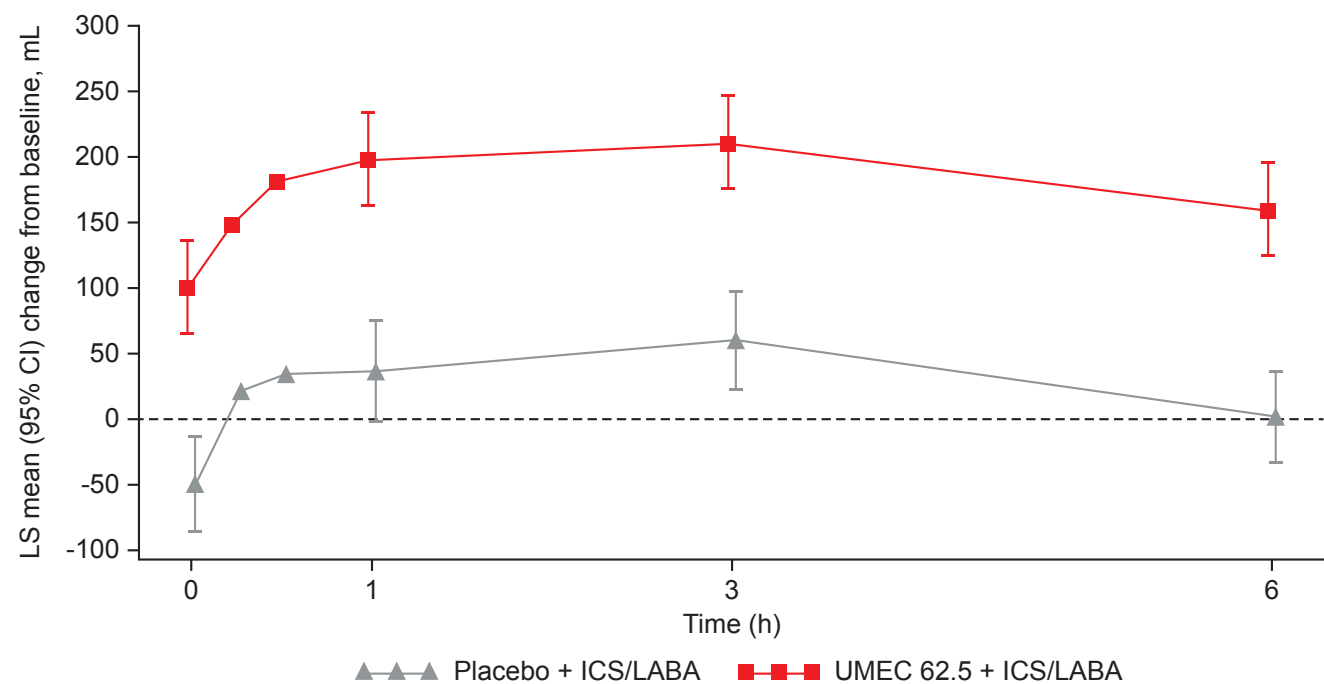

Supplement: Supplementary Figure S1 [file npjpcrm201631-s1.pdf]
