# Supplementary material for: The effect of umeclidinium added to inhaled corticosteroid/long-acting β2-agonist in patients with symptomatic COPD: a randomised, double-blind, parallel-group study
Source: NPJ Prim Care Respir Med. 2016 Jun 23;26:16031–. doi: 10.1038/npjpcrm.2016.31 (PMC4918053; doi:10.1038/npjpcrm.2016.31)
Supplement: Supplementary Figure legend [file npjpcrm201631-s2.doc]

# Supplementary file 2 – Figure legend

Figure S1. Serial FEV1 at Day 84 at the following post-dose time points: 15 min, 30 min, 1 h, 3 h, and 6 h, for the placebo + inhaled corticosteroid (ICS)/long-acting beta-agonist (LABA) and umeclidinium (UMEC) + ICS/LABA treatments groups.

**Figure S1.** Serial FEV_1_ over 0−6 h at each time point at Day 84
