# Supplementary material for: The effect of umeclidinium added to inhaled corticosteroid/long-acting β2-agonist in patients with symptomatic COPD: a randomised, double-blind, parallel-group study
Source: NPJ Prim Care Respir Med. 2016 Jun 23;26:16031–. doi: 10.1038/npjpcrm.2016.31 (PMC4918053; doi:10.1038/npjpcrm.2016.31)
Supplement: Supplementary Tables [file npjpcrm201631-s3.doc]

# Supplementary file 1 – Tables

Table S1 lists the number of patients using each of the inhaled corticosteroid (ICS)/long-acting beta-agonist (LABA) combinations, grouped according to the three sub-groups. Table S2 lists the medications which patients were prohibited from taking prior to Visit 1.

**Table S1.** The number of patients using each of the ICS/LABA combinations at baseline

| **Randomisation Strata** | **ICS/LABA Brand name (*Manufacturer*)** | **Number of subjects** |
| --- | --- | --- |
| Fluticasone propionate/ salmeterol | Seretide (*GlaxoSmithKline*) | 62 |
|  | Viani (*GlaxoSmithKline*) | 12 |
|  | Viani Forte (*GlaxoSmithKline*) | 21 |
| Budesonide/formoterol | Symbicort (*AstraZeneca*) | 102 |
| Other ICS/LABA including generics (Brand name) | FP/SAL (Atmadisc) | 5 |
|  | Budesonide/formoterol fumarate (Duoresp) | 7 |
|  | Beclometasone dipropionate/formoterol fumarate (Foster) | 8 |
|  | Budesonide/formoterol fumarate (Pulmoton) | 1 |
|  | FP/SAL (Rolenium) | 16 |
|  | Fluticasone propionate/formoterol fumarate (Flutiform) | 1 |
|  | Beclometasone dipropionate/formoterol fumarate (Inuvair) | 1 |

FP, fluticasone propionate; ICS, inhaled corticosteroid; LABA, long-acting beta-agonist; SAL, salmeterol

**Table S2.** Excluded Medications prior to Visit 1

| **Medication** | **Time interval** |
| --- | --- |
| Depot corticosteroids | 12 weeks |
| Systemic, oral or parenteral corticosteroids^1^ | 6 weeks |
| Antibiotics (for lower respiratory tract infection) | 6 weeks |
| Cytochrome P450 3A4 strong inhibitors | 6 weeks |
| ICS /LABA combination products except Seretide and approved FP/SAL 500/50 generic products, Symbicort and any other ICS/LABA (including generics) at approved doses and frequencies for COPD | 30 days |
| Seretide, approved FP/SAL 500/50 generic products, Symbicort and any other ICS/LABA (including generics) at approved doses and frequencies for COPD | 12 hours prior to screening |
| Phosphodiesterase 4 (PDE4) inhibitors (roflumilast) | 14 days |
| LAMA (tiotropium, aclidinium, glycopyrronium, umeclidinium) | 7 days |
| Inhaled LABA:  -salmeterol, formoterol  -olodaterol, indacaterol | 48 hours  14 days |
| LAMA/LABA combination products | Apply whichever mono component has the longest washout |
| Theophyllines | 48 hours |
| Oral beta-agonists   - Long-acting - Short-acting | 48 hours  12 hours |
| Inhaled short acting beta-agonists^2^ | 4 hours |
| Inhaled short-acting anticholinergics | 4 hours |
| Inhaled short-acting anticholinergic/short-acting beta-agonist combination products | 4 hours |
| Any other investigational medication | 30 days or within 5 drug half-lives (whichever is longer) |
| 1. Intra-articular corticosteroid injections were permitted. 2. Use of study provided albuterol/salbutamol was permitted during the study, except in the 4-hour period prior to spirometry testing. | |

COPD, chronic obstructive pulmonary disease; FP, fluticasone propionate; ICS, inhaled corticosteroid; LABA, long-acting beta-agonist; LAMA, long-acting muscarinic antagonist; SAL, salmeterol
